# Towards OPtimal TIming and Method for promoting sUstained adherence to lifestyle and body weight recommendations in postMenopausal breast cancer survivors (the OPTIMUM-study): protocol for a longitudinal mixed-method study

**DOI:** 10.1186/s12905-021-01406-1

**Published:** 2021-07-06

**Authors:** Sandra J. M. van Cappellen-van Maldegem, Floortje Mols, Nicole Horevoorts, Anja de Kruif, Laurien M. Buffart, Dounya Schoormans, Hester Trompetter, Sandra Beijer, Nicole P. M. Ezendam, Michiel de Boer, Renate Winkels, Ellen Kampman, Jantine Schuit, Lonneke van de Poll-Franse, Jacob C. Seidell, Meeke Hoedjes, the OPTIMUM research team

**Affiliations:** 1 CoRPS - Center of Research On Psychological and Somatic Disorders, Department of Medical and Clinical Psychology, Tilburg University, PO Box 90153, 5000 LE Tilburg, the Netherlands; 2 Netherlands Comprehensive Cancer Organisation (IKNL), Utrecht, the Netherlands; 3 Department of Epidemiology and Biostatistics, Amsterdam Public Health (APH), VUmc, Amsterdam, the Netherlands; 4 Radboudumc, Department of Physiology, Radboud Institute for Health Sciences, Nijmegen, the Netherlands; 5 Department of Health Sciences and the Amsterdam Public Health Research Institute, VU University Amsterdam, Amsterdam, the Netherlands; 6 Department of General Practice and Elderly Care Medicine, UMCG, Groningen, the Netherlands; 7 Division of Human Nutrition and Health, Wageningen University, Wageningen, the Netherlands; 8 Department of Psychosocial Research and Epidemiology, Netherlands Cancer Institute, Amsterdam, the Netherlands; 9 Department of Nutrition, Dietetics and Lifestyle, School of Allied Health, HAN University of Applied Sciences, Nijmegen, the Netherlands

**Keywords:** Postmenopausal breast cancer survivors, Body weight, Lifestyle, Stages of change, Need for support, Behavior Change Techniques, Mixed-method design, Patient reported outcomes, Biomarkers, PROFILES registry

## Abstract

**Background:**

The majority of postmenopausal breast cancer (PMBC) survivors do not adhere to lifestyle recommendations and have excess body weight. In this group, this is associated with poorer health-related quality of life and an increased risk of type II diabetes mellitus, cardiovascular disease, second primary cancers, cancer recurrences, and mortality. Gaining and maintaining a healthy lifestyle and body composition is therefore important. It is unknown when and how sustained adherence to these recommendations can be promoted optimally in PMBC survivors. Therefore, the OPTIMUM study aims to identify the *optimal timing* and *method* for promoting sustained adherence to lifestyle and body weight recommendations in PMBC survivors.

**Methods:**

The OPTIMUM-study has a mixed-methods design. To assess *optimal timing*, a longitudinal observational study will be conducted among approximately 1000 PMBC survivors. The primary outcomes are adherence to lifestyle and body weight recommendations, readiness for change, and need for support. Questionnaires will be administered at 4–6 months after cancer diagnosis (wave 1: during treatment and retrospectively before diagnosis), 1 year after diagnosis (wave 2: after completion of initial treatment), and 1.5 years after diagnosis (wave 3: during follow-up). Wave 2 and 3 include blood sampling, and either wearing an accelerometer for 7 days or completing a 3-day online food diary (randomly assigned at hospital level). To assess the *optimal method*, behavioural determinants of the primary outcomes will be matched with Behavior Change Techniques using the Behaviour Change Technique Taxonomy. Qualitative research methods will be used to explore perceptions, needs and preferences of PMBC survivors (semi-structured interviews, focus groups) and health care providers (Delphi study). Topics include perceptions on optimal timing to promote adherence; facilitators and motivators of, and barriers towards (sustained) adherence to recommendations; and acceptability of the selected methods.

**Discussion:**

The OPTIMUM study aims to gain scientific knowledge on when and how to promote sustained adherence to lifestyle and body weight recommendations among PBMC survivors. This knowledge can be incorporated into guidelines for tailored promotion in clinical practice to improve health outcomes.

## Background

A large body of evidence has demonstrated that higher levels of body fatness, adult excessive weight gain, drinking alcohol, and physical inactivity increase the risk of postmenopausal breast cancer (PMBC) [1, 2]. PMBC survivors are defined as people who are living with a diagnosis of PMBC, including those who have recovered from the disease [1]. PMBC survivors with an unfavorable lifestyle and body composition have a lower health-related quality of life (HRQoL), an increased risk for type II diabetes mellitus, cardiovascular disease, second primary cancers, cancer recurrences, and mortality [3–6]. Several biological mechanisms, such as enhanced inflammation, underlie these health-related outcomes [7–9]. To increase HRQoL and decrease the risk of the development of comorbidities and mortality [10–15], lifestyle and body weight recommendations have been issued [1, 16]. However, the majority of PMBC survivors does not meet these recommendations [1, 17–22].

Although numerous studies have shown that lifestyle interventions result in, mostly short-term, improvements in lifestyle and body weight in cancer survivors, the optimal timing and method to enhance long-term adherence to lifestyle and body weight recommendations remains unknown [23, 24]. Previous studies have used a top-down approach to promote adherence to recommendations in cancer survivors. These studies have generally applied (adapted versions of) interventions that have previously been proven effective in other populations. So far, this approach has not led to increased insight into the optimal method and timing to promote sustained adherence to recommendations in cancer survivors. Accumulation of scientific evidence is hindered by several factors. For instance, poor reporting of intervention components in the scientific literature [25, 26], and a lack of extensive process evaluations to identify effective intervention components and underlying behavior change mechanisms. Moreover, intervention studies are typically not designed to assess optimal timing of lifestyle support. In addition, these studies typically promote adherence to recommendations in those who are ready to change their lifestyle [27, 28], as intervention participants are generally ready to change their lifestyle whereas non-participants are not. Ideally, adherence should also be promoted in those not ready for lifestyle change.

For this reason, readiness for lifestyle change should be taken into account in promoting lifestyle, since each stage of change ((not ready: pre-contemplation/contemplation); (ready: preparation/action/maintenance); (relapse: relapse) [29]) requires different behavior change techniques [29–31]. Oncology health-care professionals play a key role in lifestyle-related information provision to cancer survivors. (Oncology) health care professionals may promote readiness for lifestyle change, since receiving a cancer diagnosis has been marked as a ‘teachable moment’ to promote adherence [32]. Unfortunately, lifestyle and body weight recommendations for cancer survivors are currently not well imbedded in Dutch health care. Although oncology health-care professionals play a key role in information provision to cancer survivors, they do not routinely provide information on the health benefits of meeting lifestyle and body weight recommendations (e.g., lower risk of all-cause, cancer-specific, and cardiovascular disease morbidity and mortality [33]).

In addition, a different approach of lifestyle support by (oncology) health care professionals is required for those with and without a perceived need for support for improving or maintaining a (healthy) lifestyle. For those who perceive a need for support, receiving information is not sufficient to achieve adherence, and additional support should be offered [34]. Such support should be tailored to one’s needs and preferences to promote uptake of, compliance to, and effectiveness of support [34]. Tailoring promotion of adherence to individual characteristics, is in line with current consensus on the importance of personalized care [35]. Such tailoring typically does not incorporate the variety of consequences of cancer and its treatment that may act as barriers or facilitators for lifestyle change after a cancer diagnosis. For example, impaired psychological health (e.g. depressive symptoms) is typically not taken into account while promoting lifestyle change in cancer survivors. However, impaired psychological health is relatively common up to years after a cancer diagnosis [36] and negatively related to health behaviors (e.g., being physically inactive) [37, 38]. A more holistic approach to promoting health behavior change includes incorporation of traditional health behavior change determinants (e.g., self-efficacy) as well as the barriers and facilitators related to physical and psychological health after cancer diagnosis and treatment.

In contrast with the top-down approach to promotion of health behavior change in cancer survivors used in previous studies, the OPTIMUM-study will use a bottom-up approach (i.e., building scientific evidence from basic psychosocial research, rather than from application of existing complex interventions) for individualised intervention development from knowledge on specific modifiable determinants relevant for PMBC survivors. By matching specific modifiable determinants relevant for this specific patient population to behavior change techniques, a ‘toolbox’ containing a variety of building blocks (i.e., intervention ingredients) can be composed. This toolbox can be used to create individualized interventions by selecting the right tools for each specific individual.

To accumulate scientific evidence on the optimal timing and method to promote sustained adherence to lifestyle and bodyweight recommendations in PMBC survivors, the OPTIMUM-study (Towards OPtimal TIming and Method for promoting sUstained adherence to lifestyle and body weight recommendations in postMenopausal breast cancer survivors) was initiated. The OPTIMUM-study uses a systematic, bottom-up, holistic approach [39]. The overall aim is to gain insight into the optimal timing and method to promote (sustained) adherence to lifestyle and bodyweight recommendations in (subgroups) of PMBC survivors.

The OPTIMUM study has two key objectives with several sub-objectives:

*Key objective 1*: To gain insight into the *optimal timing* to promote (sustained) adherence to lifestyle and body weight recommendations in PMBC survivors.

This is further specified into the following sub-objectives:

- 1a. To longitudinally assess proportions of PMBC survivors’ *non-adherence* and *need for support* to be able to improve lifestyle or maintain lifestyle improvements.
- 1b. To examine *socio-demographic and clinical characteristics* of those who do (not) adhere and of those who (not) indicate a need for support over time.
- 1c. To examine *biological markers* in relation to lifestyle and bodyweight of those who do (not) adhere and of those who (not) indicate a need for support over time.
- 1d. To explore perceptions on optimal timing among PMBC survivors, oncology health care professionals, and other relevant stakeholders.

*Key objective 2*: To gain insight into the *optimal method* for (oncology) health care professionals to promote (sustained) adherence to lifestyle and body weight recommendations in subgroups of PMBC survivors.

This is further specified into the following sub-objectives:

- 2a. To compose ‘*patient profiles*’ according to ‘adherence to a particular recommendation’, ‘readiness for change’, and ‘need for support’;
  - To describe which *patient profiles* are most prevalent per time point;
  - To describe *socio-demographic and clinical characteristics* of the most frequent patient profiles.
- 2b. To assess personal, clinical, and cancer-related *modifiable determinants* of *adherence*, *readiness for change*, and *need for support* in PMBC survivors over time;
  - To gain knowledge on which determinants should be targeted to promote sustained adherence;
  - To describe *modifiable determinants* of the most frequent patient profiles.
- 2c. To select Behavior Change Techniques [30] that could be used to influence the associated modifiable determinants (i.e., toolbox containing potential intervention ingredients).
- 2d. To explore the *acceptability of the selected Behavior Change Techniques*, and to explore *perceptions on the optimal method* to promote (sustained) adherence among PMBC survivors, oncology health care professionals, and other relevant stakeholders.

## Design and methods

### Design

To increase knowledge on *optimal timing* for promotion of sustained adherence in PMBC survivors, the OPTIMUM-study longitudinally assesses adherence to lifestyle and body weight recommendations, readiness for change, and need for support to be able to adhere to these recommendations over time. To increase knowledge on the *optimal method* for promotion of sustained adherence in PMBC survivors, modifiable determinants of health behavior change relevant for this specific patient population will be assessed and matched to behavior change techniques. That knowledge will be used to obtain a ‘toolbox’ of ‘building blocks’ (i.e. behavior changes techniques) that can be used in composing an individually tailored intervention for PMBC patients [30]. PMBC survivors will be categorized into ‘*patient profiles*’ according to the answers to the following questions: (1) Does one need to change their lifestyle behavior to be able to adhere to a particular lifestyle or body weight recommendation? (as assessed by *adherence* to a particular lifestyle recommendation), (2) To what extent is one ready to change her lifestyle behavior? (assessed by *readiness for change*), and (3) Is one able to achieve change by herself or does she need support to be able to improve a specific health behavior? (assessed by *need for support*). Each patient profile requires a different combination of behaviour changes techniques (building blocks) to promote health behaviour change. See “Appendix” for an overview of patient profiles.

**Fig. 1:**
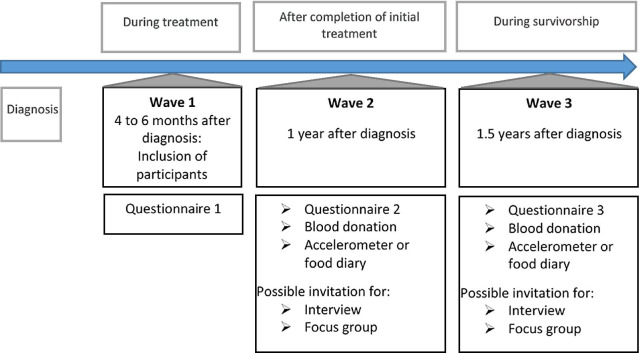
Design of the OPTIMUM-study.

The OPTIMUM-study is a longitudinal observational study with a mixed-methods design, comprising both quantitative and qualitative measurements. Quantitative measurements will include questionnaires at 4–6 months after cancer diagnosis (wave 1: during treatment, with retrospective measurement before diagnosis), 1 year after diagnosis (wave 2: after completion of initial treatment), and 1.5 years after diagnosis (wave 3: during follow-up). As additional markers of adherence, at wave 2 and 3 quantitative measurements will include blood sampling (in 9 out of 16 participating hospitals) and either wearing an accelerometer for 7 days, or completing an online 3-day food diary (randomly assigned at hospital level). Qualitative measurements will include semi-structured interviews based on purposive sampling at wave 2 and wave 3, focus groups after the interviews, and a Delphi-study. The qualitative research methods will be used to explore perceptions, needs and preferences of PMBC survivors (semi-structured interviews, focus groups) and health care providers (Delphi study). See Fig. 1 for an overview of the design of the OPTIMUM-study.

### Study population

Inclusion criteria are having been diagnosed with breast cancer 4 to 6 months ago and being postmenopausal (i.e., not having menstruated for at least 1 year). Exclusion criteria are having been diagnosed with a Ductal Carcinoma in Situ and not being able to independently understand and complete a Dutch questionnaire, or being interviewed in Dutch.

### Recruitment

Patients will be invited for study participation by their own oncology health-care professional (i.e., oncologist, internist, surgeon, or mamma care nurse) from 16 participating hospitals across the Netherlands. Eligible patients will receive an invitation letter during a visit to their oncology health care professional. After providing written informed consent, participants will be invited to complete either an online or paper version of the first questionnaire (wave 1). According to their preference for completing either an online or paper version of the questionnaire, participants will be contacted for data collection at waves 2 and 3. Participants who prefer to complete a paper version of the questionnaire will receive printed questionnaires by mail. Participants who prefer to complete an online version of the questionnaire will receive a link to the online questionnaire via e-mail. Online questionnaires will be completed via the PROFILES (Patient Reported Outcomes Following Initial treatment and Long term Evaluation of Survivorship) registry [27]. In case of non-response, one reminder will be sent per participant per wave (either via e-mail or by mail according to their preference), followed by up to 5 telephone calls. The OPTIMUM study aims to recruit approximately 1000 participants. Approximately 25 PMBC survivors will be invited for semi-structured interviews based on purposive sampling according to (non)adherence and need for support over time as assessed by means of questionnaires in wave 2 and 3.

Participants are not informed about lifestyle and body weight recommendations as part of the OPTIMUM study because of its observational nature. As such, whether or not participants are informed about the recommendations depends on the standard care they receive. Standard care for participants currently does not include information provision about lifestyle and body weight recommendations, although differences between hospitals and health care professionals do exist.

### Measurements

Table 1 provides an overview of all quantitative measures at wave 1, wave 2, and wave 3.

**Table 1:** Overview of quantitative measures and measurement instruments in the OPTIMUM-study

Variables	Instrument	Wave 1^a^	Wave 2^a^	Wave 3^a^	Objective
Sociodemographic and health-related variables					
Demographics (education, marital status, employment status)	Demographic questions	x	x	x	1b
Comorbidities	Self-administered Comorbidity Questionnaire (SCQ) [60]	x	x	x	1b
Estrogen/breast cancer related variables	Questionnaire items concerning age of onset menarche in years, number of pregnancies, total duration of breastfeeding, age of onset menopause in years	x			1b
Cancer-specific health related quality of life	European Organization for Research and Treatment Quality of Life Questionnaire (EORTC QLQ-C30) [61]		x	x	1a
Overweight and body fat distribution					
BMI	Weight in kg/(Height in m)^2^	x	x	x	1a, 2a
Hip- and waist circumference	Self-administrated measurement by use of measurement tape	x	x	x	1a, 2a
Physical activity + sedentary behavior					
Physical activity level and sedentary behavior	Physical Activity Scale for the Elderly (PASE) [42]	x	x	x	1a, 2a
Physical activity accelerometry	7-day accelerometer data (ActiGraph) [46] *Physical activity measures*: Average acceleration (AvAcc), Intensity Gradient (IG), total minutes light, moderate and vigorous physical activity per day, total minutes of inactive time per day, most active continuous 30 min (M30) per day		x	x	1a, 2a
Functional muscle strength	5Times-Sit-To-Stand functional muscle strength measurement: self-administrated measurement by use of stopwatch [50]		x	x	2b
Dietary intake					
Diet quality (including alcohol consumption)	Dutch Healthy Diet—index 15 (DHD-15), with minor adjustments [54]	x (shortened)	x	x	1a, 2a
Dietary intake: energy and macronutrients	Online 3-day food diary: registration of all foods and drinks, in portion sizes of gram/ml, they have consumed during the day using the ‘Eetmeter’ from the Dutch ‘Voedingscentrum’		x	x	1a, 2a
Smoking					
Smoking behaviours	Smoking behaviour questions	x	x	x	1a, 2a
Sleep					
Sleep quality and disturbances	Pittsburgh Sleep Quality Index (PSQI) [55]		x	x	1a, 2a
Sleep accelerometry	7-day accelerometer data (wristworn ActiGraph wGT3X) [46] *Sleep measures*: sleep latency, sleep efficiency, daytime sleep, frequency of long sleep interruptions (> 5 min), total minutes of sleep per night		x	x	1a, 2a
Lifestyle and health related measures					
Readiness for lifestyle change	Assessed according to the transtheoretical model (not ready: pre-contemplation/contemplation); (ready: preparation/action/maintenance); (relapse: relapse) [29] with 1 item per recommendation	x	x	x	2a, 2b, 2c
Need for support	Need for support assessed with 1 item per recommendation	x	x	x	1a, 2a, 2b, 2c
Posttraumatic growth	Posttraumatic Growth Inventory (PGI) [62]		x	x	2b, 2c
Self-compassion	Short Form Self-Compassion Scale [63]: 6 positive items only	x	x	x	2b, 2c
Emotion regulation	Cognitive Emotion Regulation Questionnaire (CERQ)—short [64]		x	x	2b, 2c
Mental and physical fatigue	Multidimensional Fatigue Inventory (MFI) [65]		x	x	2b, 2c
Symptoms of depression and anxiety	Hospital anxiety and depression scale (HADS) [66]		x	x	2b, 2c
Biological determinants of cancer prognosis					
Inflammation	Pro- and anti-inflammatory cytokines (TNFα, IL-6, IL-10, IL-1Ra) and CRP		x	x	1c
Metabolism	leptin, insulin, insulin growth factor-1, glucose, HbA1C, total cholesterol, triglycerides, HDL cholesterol, LDL cholesterol, Vitamin D		x	x	1c

^a^wave 1 = 4–6 months after diagnosis; wave 2 = 1 year after diagnosis; wave 3 = 1.5 years after diagnosis

Table 2 provides an overview of the study criteria used to determine (non-)adherence to the lifestyle and body weight recommendations of the World Cancer Research Fund (WCRF) [1, 2], as well as the recommendation for sleep of the American Academy of Sleep Medicine and Sleep Research Society (AASM&SRS) [16].

**Table 2:** Overview of study measures to determine (non-)adherence to the lifestyle and body weight recommendations of the World Cancer Research Fund (WCRF) [1, 2], as well as the recommendation for sleep of the American Academy of Sleep Medicine and Sleep Research Society (AASM&SRS) [16]

Lifestyle and bodyweight recommendations	Operationalization of recommendation	Measurement instrument used to assess recommendation
Weight [1]	BMI between 18.5 and 24.9 kg/m^2^*Waist circumference below 80 cm*	*Standardized questions weight and height* *Self-administered hip- and waist circumference measurement*
Physical activity [1]	*At least 150 min of low intensity exercise during 1 week, spread over several days* *At least 2 times a week muscle and bone strengthening exercises* *Prevent sitting too much and limit sedentary behavior*	*Questionnaire*: The Physical Activity Scale for the Elderly (PASE) [42] *Actigraph* (7 days): average acceleration (AvAcc), intensity gradient (IG), total minutes light, moderate and vigorous physical activity per day, total minutes of inactive time per day, most active continuous 30 min (M30) per day.(*randomized at hospital level*) [46]
Wholegrains, vegetables, fruit and beans [1]	Eat at least 250 g of vegetables each day Eat at least 2 pieces of fruit each day Eat beans at least once a week Eat at least 30 g of wholegrains each day	*Questionnaire*: Dutch Healthy Eating Index [54] *Online 3 day Food diary (randomized at hospital level)*
Fast foods [1]	Limit consumption of processed foods high in fat, starches or sugar—including fast foods: any pre-prepared dishes, snacks, bakery foods, deserts, and confectionary (candy)	*Questionnaire*: Dutch Healthy Eating Index [54] *Online 3 day Food diary (randomized at hospital level)*
Meat products [1]	Eat no more than 350 to 500 g of red or processed meat per week	*Questionnaire*: Dutch Healthy Eating Index [54] *Online 3 day Food diary (randomized at hospital level)*
Sugary drinks [1]	Drink mostly water and unsweetened drinks	*Questionnaire*: Dutch Healthy Eating Index [54] *Online 3 day Food diary randomized at hospital level)*
Alcoholic drinks [1]	Drink no alcohol	Standardized questions alcohol consumption
Smoking [2]	Do not smoke	Standardized smoking questions
Sleep [16]	Sleep at least 7 h per night	*Questionnaire*: Pittsburg Sleep Quality Index [55] *Actigraph* (7 days): sleep latency, sleep efficiency, daytime sleep, frequency of long sleep interruptions (> 5 min), total minutes of sleep per night (*randomized at hospital level*) [46]

#### Quantitative measures

- *Overweight and body fat distribution*. Excess body weight and body fat distribution will be determined by self-reported *height* and *weight* with which we calculate BMI [15] and self-measured *hip- and waist circumference* [40]*.* The waist circumference and waist/hip ratio provides an indication of body fat distribution (i.e. abdominal fat accumulation) and associated disease risk [41].
- *Physical activity and sedentary behaviour*
  *Physical activity* will be assessed with the Physical Activity Scale for the Elderly (PASE) [42], a 13-item questionnaire that assesses participation in leisure activities. In addition, muscle strengthening activities will be recorded, as well as time spent on paid or unpaid work, and household activities [43]. The PASE has shown to have good to excellent test–retest reliability, and to be a reasonably valid method to classify healthy elderly individuals and cancer patients into categories of physical activity [43–45].
  Detailed data on participants’ *physical activity and sedentary behaviours* will be collected using an accelerometer, the ActiGraph wGT3X [46]. Survivors treated in a hospital selected for wearing the accelerometer will wear an accelerometer on their wrist for 7 consecutive days on their non-dominant arm. Upon return of the ActiGraph, the data will be downloaded using the accompanying software ActiLife (Version 6.13.3; ActiGraph, Pensacola, FL, USA) and saved in raw format. Subsequently, the.gt3x files are converted to time-stamp free.csv files which could be exported into R v.3.6.0. The.csv files are processed using the R-package GGIR v.2.1-0 [47, 48]. Data of participants will be excluded from subsequent analysis if their accelerometer files demonstrated a post-calibration error bigger than 0.01 g; if there are less than 3 valid wear-days (defined as ≥ 16 h per day) [49]; or if there are no wear data present for each 15 min period of the 24 h cycle. Physical activity level will be expressed as average acceleration across the day (Eucledian Norm Minus One (ENMO), mg) [49], intensity gradient across the day (IG), average time accumulated in low intensity physical activity (LPA) per day (min/day), average time accumulated in moderate-to-vigorous physical activity (MVPA) per day (min/day), average time accumulated in vigorous physical activity (VPA) per day (min/day), time spent inactively per day (min/day), and most active continuous 30 min (M30) per day.
  *Five-Times-Sit-to-Stand (FTSTS) test)*: this test will be used to determine lower body muscle function, and may indicate sarcopenia and frailty [50]. Participants will perform this test at home using a chair and a stopwatch (included in the information package). Participants will measure the time it takes to stand up and sit down five times from a chair. This test has been found valid and reliable to assess lower body muscle function [50].
- *Dietary intake*
  *Food diary: Dietary intake* will be assessed by asking the patients to complete an online food diary (the ‘Eetmeter’, a digital tool of the Netherlands Nutrition Center) to register all foods and drinks, in portion sizes or gram/ml, they have consumed during the day [51]. At wave 2 and wave 3, patients will be asked to register their daily intake during three days: two week days and one weekend day. The Eetmeter is connected to the Dutch Food Composition Database (NEVO) [52, 53] which allows for the calculation of the quantity of daily energy, micro-, and macronutrients (i.e., fat, protein, and carbohydrate) consumption automatically.
  *Adherence to dietary guidelines: Diet quality* will be assessed by use of the Dutch Healthy Diet index-15 (DHD-15) [54]. The DHD-15 is a brief food frequency questionnaire that estimates diet quality and assesses adherence to the fifteen food-based Dutch dietary guidelines of 2015 (e.g., fruit, vegetables, wholegrain products, legumes, nuts, diary, fish, tea, fats and oils, coffee, red and processed meat, sweetened beverages and fruit juices, alcohol, and salt). Per component, the scores range from 0 to 10, resulting in a total score between 0 (no adherence) to 150 (complete adherence). The ability of the DHD-15 to rank persons on their diet quality is considered to be acceptable [54]. Several of the Dutch Dietary Guidelines are similar to the WCRF recommendations, therefore, the results of the DHD-15 will also provide insight into adherence to the WCRF recommendations.
- *Smoking*
  *Smoking* will be assessed by standardized questions on smoking habits (i.e., cigarettes/shag, cigars, pipe tobacco, and e-cigarettes). PMBC survivors will be classified in; never, ex, light, and heavy smokers.
- *Sleep*
  *Sleep quality and disturbances* will be measured using the Pittsburgh Sleep Quality Index (PSQI) [55] which assesses sleep quality and disturbances over a one-month period. Nineteen items measure seven ‘component’ scores: subjective sleep quality, sleep latency, sleep duration, habitual sleep efficiency, sleep disturbances, use of sleeping medication, and daytime dysfunction. The sum of these seven component scores add up to one global score. The total global component score ranges from 0 to 21. Higher scores indicate lower sleep quality and more sleep disturbances [55]. The PSQI is known for its good validity, it is able to discriminate good from poor sleepers. In addition, internal homogeneity and consistency (test–retest reliability) are good.
  Detailed data on participants’ *sleep pattern* will be collected by use of an accelerometer, the ActiGraph wGT3X [46]. Participants will wear the accelerometer during the night (in total 7 nights) to obtain data on: sleep duration, sleep latency, wake after sleep onset, sleep interruptions, and sleep efficiency.
- *Lifestyle and health related measures*
  For each single lifestyle recommendation, the following possible changeable determinants of adherence will be determined.
  *Readiness for lifestyle change* will be measured according to the transtheoretical model (not ready: pre-contemplation/contemplation); (ready: preparation/action/maintenance); (relapse: relapse) [29] and will be assessed for each recommendation with a single item.
    For each of the lifestyle recommendations, participants will be asked to indicate which stage of change fits their current state or their state just before diagnosis (i.e., wave 1) best with self-designed questions (see Table 1). If patients have attempted to change but could not maintain this change, they automatically relapse to a prior stage of the transtheoretical model. For this reason they will be allowed to tick boxes of two stages of change, both ‘relapse’ and either ‘precontemplation’, ‘contemplation’, or ‘preparation’ [29, 31].
  *Perceived need for support.* At all measurement points and for each specific lifestyle and body weight recommendation, participants will be asked if they are in need for support to be able to change their lifestyle and/or body weight. Also, they will be asked to specify the type of support they would prefer by use of an open-ended question.
- *Biological markers in relation to lifestyle and bodyweight.*
  Blood will be collected by venipuncture at the participants’ treating hospital. All participants, in 9 out of 16 participating hospitals, will receive a lab form in their information package (at wave 2 and wave 3). Attached to this lab form, they will receive a short questionnaire. This questionnaire contains questions concerning fasted state, medication use, and sickness at the moment of blood sampling, as these factors can have an impact on the biological markers of interest. Time of blood donation will be marked on the questionnaire. Directly after blood sampling, the serum blood sample will be allowed to clot at room temperature and will be centrifuged. The EDTA blood sample will be centrifuged at room temperature directly after blood sampling. The subtracted plasma, serum, and buffy coat samples will be processed within 2 h of collection and are stored at − 80 °C until further analyses. All procedures will be defined in a protocol to ensure standardisation over study sites. All blood samples will be transported from the laboratory at the treating hospitals to the Biobank Maastricht. Following, appropriate ELISAs and ILLUMINA analyses will take place to determine the biological markers. Blood samples will be stored in a biobank for later analysis of biomarkers. Analysis of the following biomarkers is anticipated:
  *Inflammation*. Pro- and anti-inflammatory cytokines will be determined, including Tumor Necrosis Factor-alpha (TNFα), Interleukin-6 (IL-6), Interleukin-10 (IL-10), and Interleukin-1 Receptor Antagonist (IL-1Ra), and a general marker of inflammation C-reactive protein (CRP).
  *Metabolism:* biomarkers include leptin, insulin, insulin growth factor-1, glucose, glycated haemaglobin (HbA_1_C_1_), total cholesterol, triglycerides, High-Density-Lipoprotein (HDL) cholesterol, Low-Density-Lipoprotein (LDL) cholesterol, and vitamin D.
- Clinical cancer-related variables
  Data on clinical cancer-related variables will be retrieved from the Netherlands Cancer Registry (NCR), which records clinical data of all newly diagnosed cancer patients in the Netherlands.

#### Qualitative measures

- *Interviews*
  Semi-structured interviews will be held to explore perceptions on optimal timing for support (Key objective 1) and to gain insight in possible changeable determinants of adherence to lifestyle and bodyweight recommendations. PMBC survivors will be invited for semi-structured interviews based on purposive sampling according to (non)adherence, readiness for change, and need for support over time as assessed by means of questionnaires in wave 2 and 3. The number of invited participants depends on the information that comes up during the interviews. Interviews will be guided by a topic list. Discussion topics include barriers, facilitators, and motivators for adherence to recommendations in daily clinical practice, and perceptions on optimal timing of promotion of adherence. Interviews will be audiotaped and transcribed verbatim. Transcripts from the interviews will be supplemented with field notes from the interviewer. Member checking will be performed after the interviews (i.e. returning a summary of an interview to a participant to check for accuracy and whether it resonated with their experiences) [56].
- *Focus groups*
  Focus groups will be conducted after the interviews to validate and enrich the data gathered during the interviews, to prioritize possible changeable determinants of adherence, and to further explore themes that arise during the interviews. Focus groups will be audiotaped and transcribed verbatim. Field notes from the observer will be supplemented to the transcripts. Results of each focus group will be discussed between the moderator and the observer.
- *Delphi-study*
  An iterative three-round online Delphi study will be conducted to gain insight in perceptions of medical health care professionals (i.e., mamma oncology surgeons, mamma oncology internal medics, mamma oncology nurses, oncology dieticians, oncology physical therapists, oncology psychologists), policy makers, and PMBC survivors of potential barriers and facilitators for promoting lifestyle adherence in daily clinical practice. The three rounds will be respectively used for item generation, prioritizing of items, and ranking of the items.

### Data analyses

#### Quantitative data

Descriptive statistics and Generalized Linear Mixed Models (GLMM) will be used to: (1a) longitudinally assess proportions of (non-)adherence to each recommendation, readiness for change, and the need for support, to (1b) examine sociodemographic and clinical characteristics, and to (1c) examine biological determinants, of those who do not adhere and of those in need for support over time. The relation between adherence to each recommendation and socio-demographic and non-changeable clinical characteristics will be longitudinally assessed by fitting GLMM with adherence to each recommendation (no/yes) as dependent dichotomous variable and time (wave1, wave2, wave3) and socio-demographic and clinical characteristics (age, ethnicity, socioeconomic status, marital status, stage of cancer at diagnosis, type of treatment) as independent variables. We will assess the need to include interaction terms between time and the socio-demographic and clinical characteristics. These analyses will be repeated for need for support as outcome variable. Similar analyses will be conducted for biological determinants of cancer prognosis modifiable by lifestyle and bodyweight.

With regard to aim 2a, for each single lifestyle recommendation ‘patient profiles’ will be composed by creating a cross tabulation of the variables ‘adherence to a particular recommendation’ (yes/no), ‘readiness for change’ (Not ready: pre-contemplation/contemplation; ready: preparation/action/maintenance), and ‘need for support’ (yes/no) for each time point (see “Appendix”). Based on these cross tabulations, it will be examined which patient profiles are most prevalent per time point. In addition, descriptive statistics will be used to describe changeable socio-demographic (e.g., employment, education) and clinical characteristics (e.g., tumor stage, treatment received) of the most frequent patient profiles. GLMM will be used to assess socio-demographic and clinical modifiable determinants of adherence, readiness for change, and need for support, as captured by the most prevalent patient profiles over time (aim 2b). We will assess the need to include interaction terms between time and the socio-demographic and clinical characteristics. Based on the behavior change technique taxonomy [30], the changeable socio-demographic and clinical determinants will be matched to suitable behaviour change techniques (aim 2c). Additionally, with respect to aim 2a and 2b, the composition of ‘patient profiles’, multilevel latent class modelling will be used combining adherence to all recommendations, readiness for change to each specific recommendation, and need for support for each specific recommendation, for each time point for all recommendations. The multilevel latent class model will be used to gain insight into the course of the patient profiles over time.

#### Qualitative data

Research objectives 1d and 2d, will be addressed by means of qualitative analysis. Specifically, exploring perceptions on optimal timing and method among PMBC survivors, oncology health care professionals, and other relevant stakeholders. With respect to the interviews and focus groups, a thematic analysis will be conducted as described in Braun and Clarke [57]. Transcripts will be subsequently disentangled, divided into fragments and open-coded. Codes will be categorized by subthemes and main themes. Relationships between the subthemes will be explored, to eventually cover the subthemes under the overall themes. The codes, subthemes, and themes will be discussed by two researchers until consensus is reached. Codes and (sub)themes will be structured in a code tree. The constant comparison method will be used in order to understand the differences, as well as similarities, between respondents and within each of the respondents. The main results will be discussed in the research team to enhance the robustness of the findings.

The output of the rounds of the Delphi-study (aim 1d and 2d) will be analysed (i.e., defining items, categorizing items, removal of duplicate items, calculating sum scores for prioritizing and ranking of items). Thereafter, the output will be used as input for the next round till, in consultation with the oncology medical professionals, a top rank of facilitators and barriers for lifestyle care will be created in the third round.

#### Combined data

Quantitative results obtained from the measurements and questionnaires will be combined with the qualitative results obtained from the individual interviews and focus group sessions. Together, these data sets will provide a more complete and comprehensive evaluation of optimal timing and method to enhance lifestyle in PMBC survivors (key objective 1 and 2).

### Sample size

The sample size calculation was conducted using the validated rule of thumb of a minimum of 10 participants per independent variable in the smallest group of the dichotomous outcome measure (e.g., 25% non-adherence [20, 58] vs. 75% adherence to the recommendation on alcohol intake) [59]. For aim 2b, incorporating the highest number of changeable determinants, a maximum of 16 changeable determinants will be incorporated in the analyses. Based on data on adherence to recommendations from previous studies in Dutch cancer survivors [20, 58], the largest number of participants needed to be able to detect valid associations between changeable determinants and adherence to each recommendation with inclusion of 16 independent variables is 860 for the recommendation for smoking (assuming 18.6% of women smoke) (16*10)/18.6 × 100). The required number of participants for the other recommendations are: 462 (160/34.62 × 100) for body weight; 601 (160/26.62 × 100) for physical activity; 375 (160/42.62 × 100) for foods and drinks that promote weight gain; 351 (160/45.6 × 100) for fruit intake; 580 (160/27.6 × 100) for vegetable intake; and 624 (160/25.66 × 100) for alcohol intake.

Furthermore, to be able to detect valid associations between (non-)changeable socio-demographic and clinical characteristics and the most prevalent patient profiles per time point (aim 2a and 2b), power analysis indicated a minimum of at least 1076 participants. Power analysis was based on an ANCOVA including 5 groups (expected number of main patient profiles in the cross-tabulation based on ‘adherence to a particular recommendation’, ‘readiness for change’, and ‘need for support’) and 3 covariates (e.g., stage of cancer at diagnosis), assuming a small effect for each predictor (partial eta squared = η_p_^2^ = 0.015).

### Stakeholder group

A stakeholder group will be actively involved throughout the study, in order to provide a solid basis for implementation and dissemination of study findings. This group consists of 10–15 stakeholders, including representatives of: the Dutch breast cancer patient association, professional bodies for health care professionals such as oncologists, oncology nurses, and general practitioners; policy makers, and a representative of health insurance companies. Stakeholders will be: informed about study progress, consulted for advice on issues that may arise throughout the study, involved in decision making, and in writing a plan for adoption, implementation, sustainability, and evaluation of guidelines on how and when to promote adherence. In addition, stakeholders will be consulted individually by telephone or e-mail when necessary.

### Ethical considerations

The study protocol has been reviewed and approved by the medical research ethics committee METC Brabant (Medical Research Ethics Committee Brabant, the Netherlands, reference number: NL66913.028.18). In addition, the study has been reviewed and approved by the local ethics committees of the participating centers.

### Data security/disclosure of original documents

Confidentiality and anonymity of participants will be guaranteed by assigning a study number to each participant. All collected data will all be stored in a secured location for 15 years.

## Discussion

In most PMBC patients lifestyle and bodyweight are suboptimal [1, 17–22], which may be related to unhealthy lifestyle behaviors. The OPTIMUM-study aims to provide scientific evidence on *when* and *how* to promote sustained adherence and in *which* PMBC patients. The study leads to products (i.e. a toolbox) that can be used in clinical practice to promote sustained adherence to lifestyle and bodyweight recommendations in PMBC patients.

### Trial status

The inclusion of patients started in February 2019. Patients will be followed up for 1.5 years after diagnosis. The COVID-19 pandemic has delayed the inclusion of PMBC survivors in the OPTIMUM-study.

## Data Availability

After finishing the data collection, the data will be freely available for non-commercial scientific research, subject to study question, privacy and confidentiality restrictions, and registration (www.profilesregistry.nl).

## Appendix: Overview of the categorization of cancer survivors into ‘Patient Profiles’ according to adherence to a particular WCRF-recommendation, stage of change, and perceived need for support.

**Table:**

**Stage of change** [29]		Adherence to a particular WCRF-recommendation	
		Does not meet recommendation	Meets recommendation
*Not ready for change*			
*Precontemplation* (*not ready*): not intending to take action in the next six months	No need for support	Cancer survivors who do not meet a WCRF-recommendation, do not intend to change their behavior, and do not perceive a need for support	Cancer survivors who meet a WCRF-recommendation, do not intend to change their behavior, and do not perceive a need for support
	Need for support	Cancer survivors who do not meet a WCRF-recommendation, do not intend to change their behavior, and perceive a need for support^a^	Cancer survivors who meet a WCRF-recommendation, do not intend to change their behavior, and report a perceived need for support^a^
*Contemplation* (getting ready): intending to take action in the next 6 months	No need for support	Cancer survivors who do not meet a WCRF-recommendation, intend to change in the foreseeable future, and do not perceive a need for support	Cancer survivors who meet a WCRF-recommendation, intend to change in the foreseeable future, and do not perceive a need for support
	Need for support	Cancer survivors who do not meet a WCRF-recommendation, intend to change in the foreseeable future, and perceive a need for support	Cancer survivors who meet a WCRF-recommendation, intend to change in the foreseeable future, and perceive a need for support
*Ready for change*			
*Preparation (ready)*: ready to take action in the next 30 days	No need for support	Cancer survivors who do not meet a WCRF-recommendation, intend to take action in the immediate future, and do not perceive a need for support	Cancer survivors who meet a WCRF-recommendation, intend to take action in the immediate future, and do not perceive a need for support
	Need for support	Cancer survivors who do not meet a WCRF-recommendation, intend to take action in the immediate future, and perceive a need for support	Cancer survivors who meet a WCRF-recommendation, intend to take action in the immediate future, and perceive a need for support
*Action*: has made overt lifestyle changes in the past 6 months	No need for support	Cancer survivors who do not meet a WCRF-recommendation, but have made overt lifestyle changes in the past 6 months, and do not perceive a need for support	Cancer survivors who meet a WCRF-recommendation, have made overt lifestyle changes in the past 6 months, and do not perceive a need for support
	Need for support	Cancer survivors who do not meet a WCRF-recommendation, but have made overt lifestyle changes in the past 6 months, and who perceive a need for support (to maintain lifestyle changes)	Cancer survivors who meet a WCRF-recommendation, have made overt lifestyle changes in the past 6 months, and who perceive a need for support (to maintain lifestyle changes)
*Maintenance*: doing a new behavior for more than six months	No need for support	Cancer survivors who do not meet a WCRF-recommendation, but have been maintaining lifestyle changes for at least 6 months, and who do not perceive a need for support	Cancer survivors who meet a WCRF-recommendation, have been maintaining lifestyle changes for at least 6 months, and who do not perceive a need for support
	Need for support	Cancer survivors who do not meet a WCRF-recommendation, but have been maintaining lifestyle changes for at least 6 months, and who perceive a need for support to maintain their changes	Cancer survivors who meet a WCRF-recommendation, have been maintaining lifestyle changes for at least 6 months, and who do not perceive a need for support to maintain their changes

^a^Unlikely scenario, expected cell-frequency of near zero.

## Abbreviations

OPTIMUM: Towards OPtimal TIming and Method for promoting sUstained adherence to lifestyle and bodyweight recommendations in postMenopausal breast cancer survivors
PMBC: Postmenopausal breast cancer
HRQoL: Health-related quality of life
PROFILES: Patient Reported Outcomes Following Initial treatment and Long term Evaluation of Survivorship
WCRF: World Cancer Research Fund
AASM&SRS: American Academy of Sleep Medicine and Sleep Research Society
PASE: Physical Activity Scale for the Elderly
DHD-15: Dutch Healthy Diet index-15
PSQI: Pittsburgh Sleep Quality Index
SCQ: Self-administered Comorbidity Questionnaire
PGI: Posttraumatic Growth Inventory
SCS-SF: Self-Compassion Scale—Short Form
MFI: Multidimensional Fatigue Inventory
HADS: Hospital Anxiety and Depression Scale
EORTC QLQ-C30: European Organisation for Research and Treatment of Cancer Quality of Life Questionnaire
AvAcc: Average acceleration
IG: Intensity Gradient
M30: Most active continuous 30 min per day
FTSTS: Five-Times-Sit-To-Stand
NEVO: Dutch Nutrients Database
TNFα: Tumour Necrosis Factor alpha
IL-6: Interleukin-6
IL-10: Interleukin-10
IL-1Ra: Interleukin-1 Receptor Antagonist
CRP: C-reactive protein
HbA1C1: Glycated haemaglobin
HDL-cholesterol: High-Density-Lipoprotein cholesterol
LDL-cholesterol: Low-Density-Lipoprotein cholesterol
DHEAS: Dehydroepiandrosterone sulfate
NCR: Netherlands Cancer Registry
GDPR: General Data Protection Regulation
IKNL: Netherlands Comprehensive Cancer Organisation
